# Emerging role of AMPA receptor subunit GluA1 in synaptic plasticity: Implications for Alzheimer's disease

**DOI:** 10.1111/cpr.12959

**Published:** 2020-11-13

**Authors:** Wenrui Qu, Baoming Yuan, Jun Liu, Qianqian Liu, Xi Zhang, Ranji Cui, Wei Yang, Bingjin Li

**Affiliations:** ^1^ Department of Hand Surgery The Second Hospital of Jilin University Changchun China; ^2^ Jilin Provincial Key Laboratory on Molecular and Chemical Genetic The Second Hospital of Jilin University Changchun China; ^3^ Department of Orthopedics The Second Hospital of Jilin University Changchun China; ^4^ Department of Burn Surgery The First Hospital of Jilin University Changchun China

**Keywords:** Alzheimer's disease, AMPA receptors, GluA1, memory, synaptic plasticity

## Abstract

It is well established that GluA1 mediated synaptic plasticity plays a central role in the early development of AD. The complex cellular and molecular mechanisms that enable GluA1‐related synaptic regulation remain to fully understood. Particularly, understanding the mechanisms that disrupt GluA1 related synaptic plasticity is central to the development of disease‐modifying therapies which are sorely needed as the incidence of AD rises. We surmise that the published evidence establishes deficits in synaptic plasticity as a central factor of AD aetiology. We additionally highlight potential therapeutic strategies for the treatment of AD, and we delve into the roles of GluA1 in learning and memory. Particularly, we review the current understanding of the molecular interactions that confer the actions of this ubiquitous excitatory receptor subunit including post‐translational modification and accessory protein recruitment of the GluA1 subunit. These are proposed to regulate receptor trafficking, recycling, channel conductance and synaptic transmission and plasticity.

## Graphical abstract

GluA1 mediated synaptic plasticity plays a central role in the development of AD. Amyloid beta/Tau protein has been shown to impair GluA1 and cause synaptic dysfunction and acute neurotoxicity, respectively

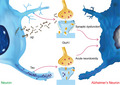

## 1 INTRODUCTION

Alzheimer's disease (AD) is a leading cause of age‐related dementia in the developed world. Deficits in synaptic plasticity, defined as any experience (activity)‐dependent changes that occur between neurons that alter their communication dynamics over time, have been a hallmark of the early aetiology of AD and similar age‐related cognitive disorders.^1^, ^2^, ^3^, ^4^ Excess amyloid‐β (Aβ) has been demonstrated to induce dysregulation of excitatory glutamate receptors like *N*‐Methyl‐d‐aspartic acid or *N*‐Methyl‐d‐aspartate receptors (NMDARs) and α‐amino‐3‐hydroxy‐5‐methyl‐4‐isoxazolepropionic acid receptors (AMPARs), one of the earliest steps in the pathogenesis of AD.^5^, ^6^, ^7^, ^8^, ^9^, ^10^, ^11^ Synaptic modulations that lead to age‐related neurodegenerative disease may occur through molecular changes such as post‐translational modifications (PTMs), protein interactions and, ultimately, structural changes at either the pre‐, post‐ or trans‐synaptic regions.^12^, ^13^, ^14^ Thus while neuron morphology may remain relatively stable over time, the structural plasticity of individual neurons may be sufficient to alter mammalian circuits such as those required for long‐term memory or cognition.^15^ Indeed, dysmorphia of neuronal microstructures and the distribution of AMPAR subunits in the synapse have all been linked to AD specifically, as detailed below.

At the memory epicentre, the hippocampus, AMPARs make up a large proportion of the excitatory synapse, up to 80% in the CA1 region.^16^, ^17^ AMPARs form tetramers of various combinations of four subunits (GluA1‐4). In hippocampal CA1 neurons, the majority of AMPARs are made up of GluA1/GluA2 and GluA2/GluA3 heterotetramers, with a small presence of GluA1 homomers and an even smaller proportion of GluA1/GluA3 heterotetramers.^18^, ^19^, ^20^ GluA1 and GluA4 contain long cytoplasmic tails, while GluA2 and GluA3 have shorter cytoplasmic tails. The composition of the receptor tetramer largely determines the functionality of the receptor. Consequently, the synaptic function is shaped by the collective receptor population. For example, some evidence has suggested that AMPARs with longer cytoplasmic tails are primarily targeted to synapses in response to neuronal activity, such as long‐term potentiation (LTP) induction, while short‐tailed receptors are constitutively targeted to synapses.^21^, ^22^, ^23^, ^24^, ^25^

AMPAR subunits are highly susceptible to regulation through a myriad of PTMs conferring changes to protein interactions, each distinctly altering the properties and function of the receptor.^25^ The layers of modulation that occur through subunit composition, protein interactions, PTMs^26^ and auxiliary subunits have been dubbed the ‘AMPAR code’ by Diering and Huaganir 2018, suggesting that as our understanding of these modulatory factors grows, the status of synaptic plasticity can be predicted.^27^ Post‐translational modification of the AMPAR subunits has also been correlated with receptor trafficking, insertion and overall abundance at the synapse.^28^ While channel properties can also be affected by a variety of PTMs (ie conductance), it is more likely that functional receptor changes such as LTP and long‐term depression (LTD) occur through changes in AMPAR abundance at the synapse.^29^ Changes in synaptic strength can trigger changes in the structure and abundance of components such as dendritic spines and axonal boutons.^15^, ^30^, ^31^ Pathological structural alterations in turn can form the basis of various diseases such as Alzheimer's, Parkinson's, schizophrenia and epilepsy.^32^, ^33^, ^34^, ^35^

**Figure 1:**
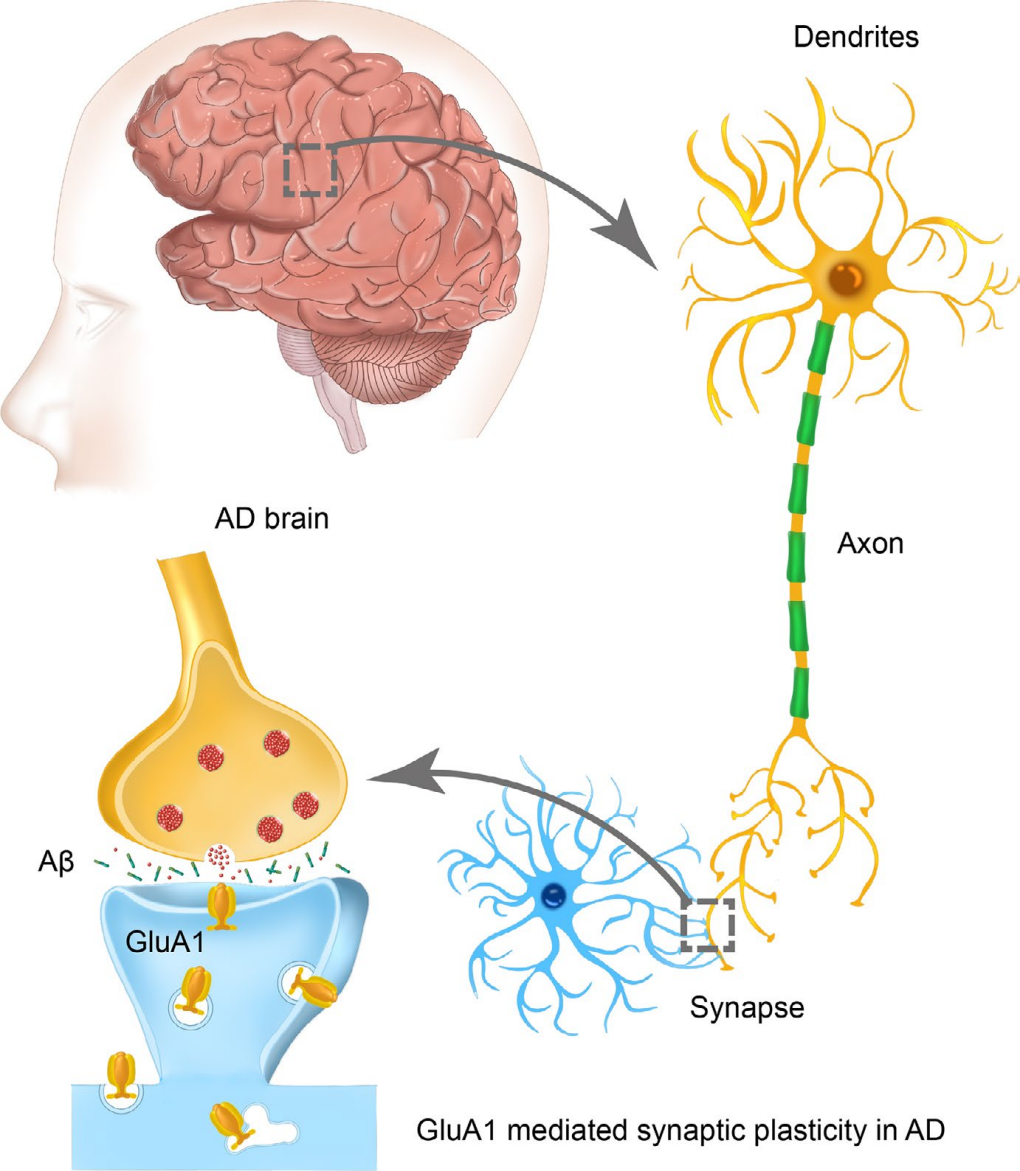
GluA1 mediated synaptic plasticity in AD. In AD brain, deficits in synaptic plasticity that occur between neurons, which alter their communication dynamics, have been the aetiology of AD. GluA1 has been linked to AD through synaptic plasticity

In this review, we outlined the role of aberrant synaptic plasticity in the aetiology of AD (predominantly amyloid β‐driven and to a lesser extent tauopathy‐driven) and dived into the role of GluA1 in synaptic plasticity, in the hippocampus as it pertains to induction and maintenance of LTP and LTD (Figure 1). We detailed the critical aspects of GluA1, such as trafficking and internalization of the subunit‐containing receptors, microstructural adaptations, changes to channel properties and PTMs that alter the interaction of GluA1 with kinases, accessory proteins and other regulatory proteins.

## 2 ROLE OF GLUA1 IN ABERRANT SYNAPTIC PLASTICITY IN DISEASE STATES

Synaptic plasticity is an essential component for learning and memory functions, endowing the ability for progressive modulations of a synapse in response to stimuli.^36^, ^37^ LTP refers to the likelihood of activation of NMDARs and subsequent calcium influx that occurs with repeated synaptic activation. Modulations like LTP lead to lasting changes in gene expression and the expression of synaptic proteins like kinases and post‐translational modifiers which propagate the modified synaptic function. Similarly, LTD occurs via low‐frequency stimulation to modulate synaptic strength in the opposite manner, reducing the likelihood of excitatory receptor activation. It is unsurprising then that a hallmark of many neurological diseases is synaptic dysfunction. For example, dysregulation of synaptic vesicle release machinery and age‐related decline in synaptic proteins have been identified as the source of cognitive impairment in animal models of ageing and AD.^38^, ^39^, ^40^, ^41^ On the other hand, pathological triggers such as cerebral infarct or formation of amyloid plaques are positively correlated with spine turnover and neurite plasticity/dysmorphia.^15^, ^42^

Alzheimer's brains are often characterized by the presence of Aβ peptides plaques, aggregated tau protein, or neurofibrillary tangles.^43^, ^44^, ^45^ The appearance of Aβ plaques often follows the appearance of Aβ oligomers, which are generally correlated with the onset of cognitive impairment, an early symptom of AD.^46^ One of the earliest molecular signs of AD is deficits in synaptic AMPAR distribution and impaired LTP/LTD.^47^, ^48^, ^49^, ^50^, ^51^ As such, special attention has been given to explorations of the role of synaptic dysfunction across various models of AD. For example, in an APP23 mouse model of age‐related Aβ accumulation, researchers found that working memory task stimulation led to a rapid decay of LTP, though no structural changes were observed.^52^

**Figure 2:**
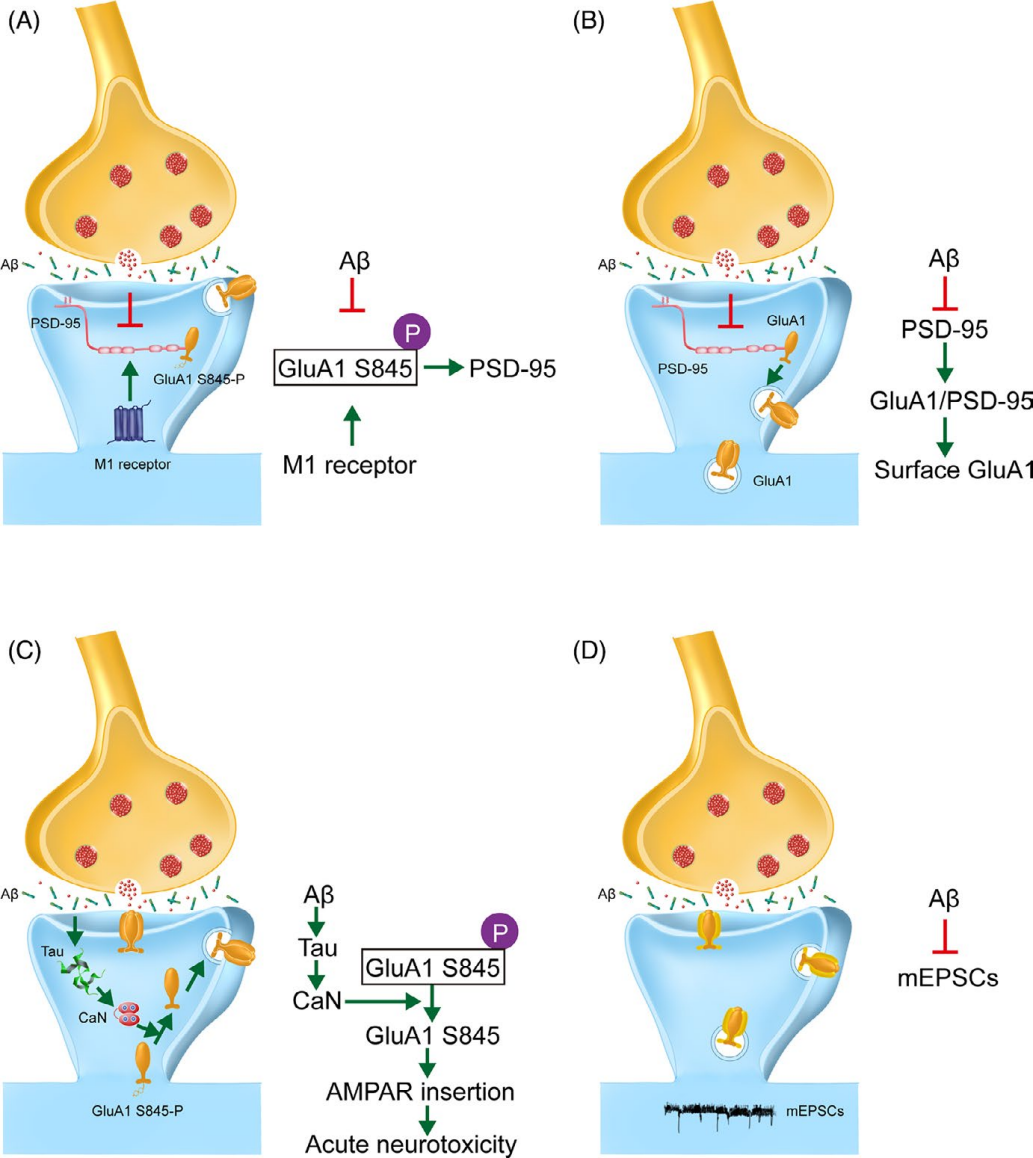
Alzheimer's related modulation of GluA1 and consequent pathology. A, M1 receptor activation can rescue cognitive impairment through modulation of GluA1 S845 phosphorylation and downstream incorporation with PSD‐95, a pathway that is compromised by Aβ aggregation. B, Aβ reduces PSD‐95, a protein involved in recruiting and anchoring glutamate receptor subunits to the post‐synaptic density. In agreement, we observed early reductions in surface expression of glutamate receptor subunit GluA1. C. Aβ oligomers cause mislocalization of tau protein to the dendritic spines. There, calcineurin‐mediated dephosphorylation of GluA1 S845 triggers a pathological cascade or rapid AMPAR insertion and acute neurotoxicity. D, Aβ oligomers impair synaptic function by decreasing the amplitude of mEPSCs

**Figure 3:**
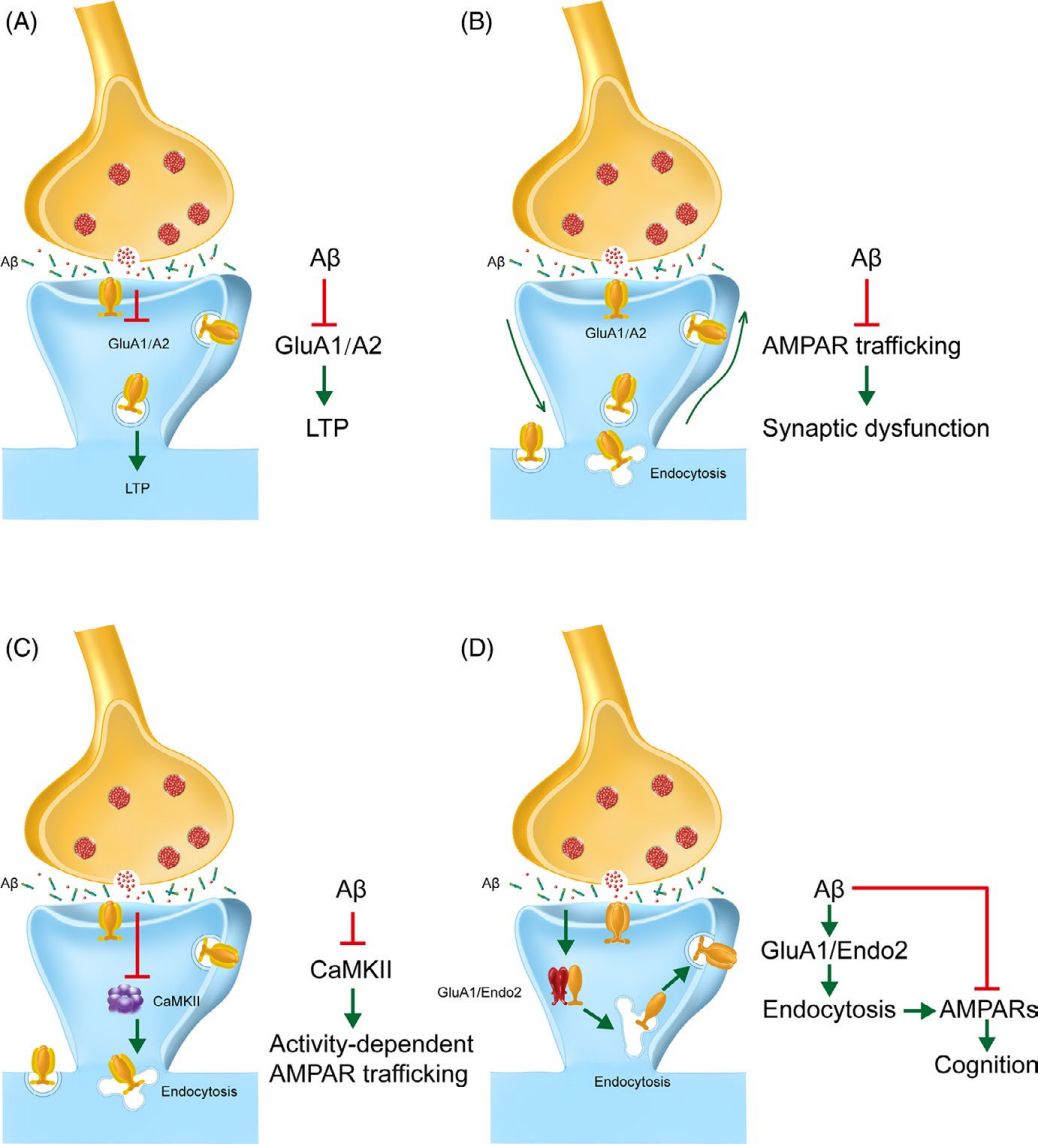
Alzheimer's related modulation of GluA1 and subsequent pathology. A, Aβ has been shown to impair synaptic plasticity through impairments in GluA1/GluA2‐mediated LTP. B, Aβ has been shown to impair AMPA trafficking and synaptic plasticity. C, Aβ interferes with CaMKII activity, disrupting activity‐dependent AMPAR trafficking. D, The loss of post‐synaptic AMPARs is mediated through the clathrin‐dependent endocytosis pathway. Endophilin 2 colocalizes and interacts with GluA1to regulate oligomeric Aβ‐induced AMPAR endocytosis

Researchers believe that a major contributor to synaptic dysfunction in AD is the disruption of AMPAR trafficking by Aβ oligomers.^53^, ^54^ Specifically, it has been reported that the Aβ oligomers may directly bind GluA2‐containing complexes,^55^ inducing acute increases in calcium‐permeable (CP) AMPARs and excitotoxicity.^56^ Aβ can also interfere with CaMKII activity, disrupting phosphorylation‐dependent AMPAR trafficking and causing subsequent deficits in LTP/LTD.^57^ The post‐synaptic protein Bin1 has been identified as a late‐onset Alzheimer's disease‐associated protein. Located in synaptic spines, reductions in Bin1 in AD models reduce exocytosis recycling, causing a build‐up of recycling endosomes, suggesting a regulatory role in trafficking. Indeed, Bin1 interacts with Arf6 and GluA1 to modulate the expression of AMPARs in the synapse.^58^ In a similar vein, various actin‐binding proteins that regulate the cytoskeleton are reduced in the hippocampi of 3xTg‐AD, contributing to altered synaptic spine morphology and function.^59^ One report proposes that age‐related increases in GluA1 subunit ubiquitination deregulate AMPAR trafficking and internalization may be an underlying mechanism of AD.^60^ In vitro, Tg2576 neurons secrete elevated levels of Aβ similar to that observed in vivo. In these cells, PSD‐95 levels are reduced in tandem with a reduction in surface expression of GluA1‐containing AMPARs,^38^ supporting the role of synaptic dysfunction in the early progression of AD. These and other proposed AD pathways are illustrated in Figures 2 and 3.

Another potential mechanism of AD pathology is homeostatic synaptic scaling, the process by which synaptic strength is slowly modified to regulate the excitability of a neuron. Typically, this involves insertion, deletion and changes in the turnover of functional receptors to maintain neurons functioning within a physiological range. However, disruptions in this mechanism can destabilize synapses and, consequently, neuronal function. Using mice that carry a double knock‐in mutation for the human gene presenilin‐1 (amyloid precursor protein), investigators demonstrated that AMPAR‐mediated (evoked and spontaneous) miniature currents are downscaled in an age‐related manner. Electron microscopy and immunohistochemistry confirmed the loss of AMPARs selectively at the CA1 stratum radiatum axo‐spinous synapses. Further, and functional tests further revealed the deficits in LTD/LTP and memory flexibility.^61^ Another study of synthetic Aβ overexpression in primary hippocampal culture resulted in an aberrant up‐regulation of AMPAR currents and cell surface expression, particularly in CP‐AMPARs through GluA2‐containing AMPARs were also affected.^62^ One study reported that in presenilin‐1 mutants, calcium signalling is abnormally elevated, as demonstrated in DIV14 cultured mutant hippocampal neurons transfected with GCaMP5, a genetically encoded Ca^2^ ^+^ indicator. Further, they found that calcineurin was also elevated in these mutants. Elevated calcineurin subsequently decreased GluA1 phosphorylation at the S845 site and selectively decreased synaptic GluA1. Interestingly, pharmacological inhibition of calcineurin with the selective inhibitor FK506 reduced elevated calcineurin in the presenilin mutant neurons, inducing synaptic scaling and selective GluA1 trafficking to the synapse.^63^ It is important to note that there are different types of amyloid plaques, and those soluble Aβ peptides appear to play the most critical roles in AD pathogenesis. Additionally, Aβ elevation is modelled differently across experimental studies; for example, in many studies, soluble Aβ is injected intracranially wherein other studies Aβ is manipulated through genetic ablation of proteins involved in Aβ production. Regardless of the model, it appears that inhibited LTP and enhanced LTD are common features of early‐onset AD pathology that precedes amyloid plaques and neural degeneration, indicating that abnormal plasticity is responsible for much of the working memory loss in early AD.^64^

Interestingly, emerging studies have elucidated a possible role for insulin resistance in the aetiology of AD and associated cognitive deficits.^41^, ^65^, ^66^, ^67^ Defective insulin signalling in the brain is a hallmark of AD, and several studies have demonstrated improved cognition and memory performance in aged and AD patients upon insulin administration.^68^ Insulin promotes trafficking of GluA1‐containing AMPARs, which appears regulated by phosphorylation of S845. Thus, reductions in hippocampal insulin or insulin receptors could suppress hippocampal LTP. In animal models of the high‐fat diet, palmitic acid is enhanced in the hippocampus leading to hippocampal insulin resistance to overexpression of the palmitoyltransferase zDHHC3. Consequently, GluA1 is hyperpalmitoylated and trafficking to the plasma membrane is inhibited. This phenomenon is concurrent with reduced AMPAR current amplitude, LTP and hippocampal‐dependent memory.^69^, ^70^

Progress in our understanding of the role of GluA1 and GluA1‐containing AMPARs in the brain will undoubtedly shed light on novel therapeutic strategies for the treatment of dementia and age‐related cognitive disorders like AD. For example, activation of M1 receptors promotes the membrane insertion of GluA1 through phosphorylation of S845, a critical site for AMPAR trafficking to the synapse.^71^ In that animal model, M1 receptor activation reversed learning and memory impairments through the modulation of GluA1 trafficking. In another study, the genetic ablation of endophilin2 (a regulatory of synaptic vesicle endocytosis) demonstrated resistance to oligomeric Aβ‐induced AMPAR dysfunction.^72^ This study highlighted that the genetic silencing of endophilin2 interacts with AMPARs to regulate oligomeric Aβ‐mediated AMPAR endocytosis in primary hippocampal culture, indicating a possible avenue for therapeutic exploration. In the Tg2576 AD animal model, 7,8‐dihyddroxyflavone, a selective TrkB agonist, increases synaptic GluA1 and GluA2 and protects against dendritic loss and preserving spatial memory despite no apparent changes to the accumulation of Aβ.^73^ Agonist‐mediated TrKB receptor activation suggests a therapeutic role of TrKB in the AD brain.

An emerging concept in Alzheimer's disease therapeutics is that sub‐toxic levels of endogenous and exogenous agents that induce oxidative stress may actually precondition the cell to future exposures such that beneficial pathways (maintenance and repair mechanisms) are triggered in response to the low‐threshold insult. In C. elegans, olive oil‐derived polyphenols demonstrated the ability to modulate stress response mechanisms to reduce the degradation of dopaminergic neurons.^74^ The dose‐dependent neuroprotective response of these plant polyphenols likely exert their hormetic effects by activating the Nr2f antioxidant response element, releasing detoxifying enzymes and positively regulating vitagenes like the longevity gene DAF‐16.^75^ In models of AD, exposure of sub‐toxic levels of hydrogen peroxide and superoxide can trigger calcium release from the endoplasmic reticulum and enhance LTP in CA1 neurons, respectively. This response to mitochondrial ROS may indicate an adaptive stress response stemming from the regulation of transcription factors aimed at protecting the mitochondria from further oxidative damage.^76^ Hormetic agents such as hydroxytyrosol (HT) not only act on the oxidative stress pathway but also can exert anti‐inflammatory and anti‐apoptotic properties as well. For example, the HT derivative HD has been shown to inhibit the nuclear translocation of NFkB in addition to reducing iNOS levels and reducing the level of pro‐apoptotic Bax. This regulation of redox homeostasis ultimately led to preservation of dopaminergic neurons in HD‐treated mice and the prevention of the pathogenic accumulation (proteotoxicity) of alpha synuclein.^77^ Beyond plant polyphenols, naturally occurring, well‐tolerated substances have been evaluated as hormetic agents in clinical trials. For example, a mushroom preparation was used experimentally in patients with Meniere's Disease (MD), a condition of cochlear neurodegeneration. In this study, nutritional supplementation activated a host of vitagenes (including HO‐1, SIRT‐1 and others) and increased ratio‐reduced glutathione in plasma.^78^ Similarly, in PC‐12 cells, application of herbal extract *Hericium Erinaceus* protected against di(2‐ethylhexyl)phthalate (DEHP)‐induced cell death. This hermetic action demonstrated both a stabilizing effect on mitochondrial membrane potential (due to reduction of intracellular ROS) and the activation of vitagenes regulated Nr2f.^79^ Identifying and exploiting neuroprotective properties of safe and well‐tolerated hormetic agents for protection against amyloid beta aggregation, or more fundamentally for the biophysical modulation of AMPA receptors is the new therapeutic frontier in the wake of limited progress with anti‐inflammatory treatments for AD. Next, we dive into multiple aspects of the AMPAR subunit GluA1 which much of the AD literature has implicated.

## 3 FUNCTIONAL PROPERTIES OF GLUA1

α‐amino‐3‐hydroxy‐5‐methyl‐4‐isoxazolepropionic acid receptors mediate the majority of excitatory activity in the brain and are essential for learning and memory. AMPARs are highly mobile and undergo both constitutive and activity‐dependent trafficking to the synapse as well as recycling and degradation. Synapse function is dictated largely by the number, localization and subunit composition of AMPARs. GluA1‐containing AMPARs account for the majority of synaptic AMPARs in the hippocampus and are the only subunit capable of forming CP homotetramers.^80^ In addition to activity‐dependent changes in receptor abundance or microstructural state, modulation of GluA1‐containing receptors can yield changes in the functional properties of the single channel. For example, GluA1 phosphorylation can increase AMPAR currents. Conversely, LTP can induce phosphorylation‐driven increases in single‐channel conductance of AMPARs.^81^, ^82^

Additional evidence of the functional changes of GluA1‐containing receptors during synaptic plasticity is taken from GluA1 gene deletion studies in mice. One study found that GluA1 deletion generated impairments in spatial working memory though it enhanced spatial memory over a 24‐hr period (long‐term). These observations suggest that GluA1 plays a differential role in short‐term and long‐term memory.^83^ Certainly, knockout of GluA1 inhibits the ability to generate LTP, a function that can be rescued in vivo with as little as 10% expression of recombinant GluA1. Below we described some of the mechanisms by which functional changes are achieved on GluA1‐containing receptors.

Much of AMPAR function is shaped by the subunit type availability at the synapse. GluA2‐containing AMPARs are continuously delivered to the spine in basal states. In contrast, GluA1‐containing AMPARs are enhanced in the synaptic surface following stimulation, first in the dendritic region followed by movement into the spines.^84^, ^85^

It is widely accepted that LTP in the hippocampus requires an increase in AMPARs within the post‐synaptic density (PSD).^86^, ^87^ A large component of AMPAR abundance is the stabilization of cell surface and synaptic expression that occurs via transmembrane AMPAR regulatory proteins (TARPs).^27^ Trafficking of GluA1‐containing AMPARs is largely driven by post‐translational modification signals that recruit kinases and accessory proteins to induce translocation to the PSD by exocytosis and lateral movement within the plasma membrane.^88^ The interaction of GluA1 and 4.1N regulates the insertion of AMPARs and the reserve pool of AMPARs required for recruitment to the synapses during LTP.^89^ Specifically, GluA1 interaction with the scaffolding protein 4.1N promotes exocytosis of GluA1 from intracellular endosomes.^89^, ^90^ On the other hand, calcium‐mediated activation of CaMKII/PKC phosphorylates GluA1 831 to promote targeting of the subunit to the PSD^81^, ^91^, ^92^, ^93^ and phosphorylation of S845 promotes GluA1 targeting to the cell surface.^94^, ^95^, ^96^ Further evidence of the importance of PTMs to the distribution of AMPARs comes from knock‐in mutation studies of the GluA1 phosphorylation sites 831/845 wherein partially impaired hippocampal LTP^97^ and inhibited delivery of GluA1 to the synapse were detected after CaMKII activation or LTP. Interestingly, double mutations do not impair LTP in the CA1 region, indicating the two sites may have a synergistic relationship on LTP expression.^98^ Of the two, S845 phosphorylation seems to be involved in GluA1 targeting/stabilization to the cell surface, likely through GluA1 recycling and limiting endocytosis.^95^, ^96^, ^99^ Finally, during hippocampal LTP, PKC mediated phosphorylation of GluA1 S818 may interact with 4.1N to maintain GluA1 exocytosis and propagate LTP as evidenced by deteriorating LTP signals in the presence of ph‐GluA1 S818 mimics and 4.1N knockdown.^89^, ^100^

During LTD, CP AMPARs (GluA1 homomers residing in the extrasynaptic region) are transiently recruited to the synapse via phosphorylation of GluA1 S845 by PKA.^101^, ^102^, ^103^ Newly incorporated CP receptors could magnify the calcium influx at synapses, resulting in LTP stabilization during the first minutes of potentiation. During the induction of LTD, these CP AMPARs signal their own removal through activation of calcineurin (CaN).^104^ Low calcium influx stimulation can activate high‐affinity phosphatases like CaN, to dephosphorylate PSD proteins. Other studies of knock‐in mutations in the GluA1 CaMKII and PKA sites exhibit deficits in LTD, indicating that dephosphorylation is important for LTD.^97^, ^105^

Like the aforementioned 4.1N, AMPAR trafficking is regulated by a myriad of accessory proteins, and deficits in the expression of these proteins lead to aberrant AMPAR trafficking and synaptic expression.^106^ For example, SAP97 (a member of the PSD‐95 family) abundance directly influences the amount of synaptic AMPARs through the binding of the GluA1 C‐terminal PDZ domain.^90^ Similarly, PSD‐95 binds GluA1 at the PDZ domains (of the N terminal) to mediate the insertion of AMPARs at synapses during LTP.^107^ Single‐particle tracking has allowed the visualization of decreased lateral mobility of GluA1‐containing AMPARs in response to auxiliary proteins like stargazing and PSD‐95, a mechanism that is thought to facilitate ‘trapping’ of AMPARs at the synapses.^108^ Other work further demonstrated that the first intracellular loop domain (Loop1) of the C‐tail of GluA1 is particularly involved in targeting AMPARs to the synapse though not for the trafficking of receptors to the plasma membrane. The authors report that CaMKII phosphorylation of S567 is a key regulator of Loop‐1‐mediated AMPAR trafficking.^109^ Finally, in addition to stabilization and recruitment of GluA1 for AMPAR induction, increases in synaptic GluA1 can be accomplished via sourcing from pre‐existing surface populations, increases in exocytosis of AMPARs, or recycling endosomes that are trafficked to the synapse following LTP.^110^, ^111^, ^112^

While it has been hypothesized that age‐related deficits in AMPAR subunit trafficking and instability may reorganize the synaptic structure and underlie age and disease related changes in cognitive ability, little direct evidence is currently available. Similarly, it has been proposed that the presence of Aβ may increase AMPAR degradation, leading to synaptic decline. Particularly, the exogenous application of Aβ activates NMDARs and triggers the removal of AMPARs.^47^ This is consistent with reports of declining AMPAR function in aged subjects. In addition to Aβ, there have been reports that hyperphosphorylated tau protein can accumulate in dendritic spines, dysregulating AMAR trafficking,^113^, ^114^, ^115^ likely due to impairments in microtubules.

## 4 GLUA1 AND STRUCTURAL ADAPTATIONS

α‐amino‐3‐hydroxy‐5‐methyl‐4‐isoxazolepropionic acid receptors can arrive at the synapse by insertion into the plasma membrane followed by lateral diffusion to the synapse.^116^ Additionally, de novo synthesis of GluA1‐containing AMPARs can occur through mRNA and translation machinery present at the dendrites, supplying AMPARs.^106^ Though the overall structure of the brain is generally unchanged in response to synaptic plasticity, microstructural changes have been documented. For example, increases in the synaptic insertion of GluA1 (such as occurs after LTP stimulus) can lead to increases in spine size in the hippocampus. In a two‐photon microscopy analysis of AMPAR trafficking during whisker stimulation, investigators found a positive correlation between GluA1 intensity at the spine and shaft and spine size.^117^ Newly synthesized GluA1 is recruited to the spines inducing increases in CA1 spine density.^118^ This structural change (attributed to the incorporation of the cytoplasmic tail of GluA1 specifically) is proposed to drive both the structural stabilization which permits spine growth and increased synaptic strength via its ligand‐gated ion channel.^119^ Interestingly, incorporation of the cytosolic fragment of GluA1 to the post‐synaptic density is sufficient to permit spine enlargement.^119^ In fact, the C‐terminal domains of AMPARs are reported to be the principal mediators of fast excitatory synaptic transmission, and genetic ablation showed that the CTD of GluA1 was critical for NMDAR‐dependent LTP but not NMDAR‐dependent LTD.^120^ AMPAR mobility can be further regulated by neuronal activity or interaction with scaffold proteins.^106^

Aβ induces synaptic aberrations by altering the morphology and composition of synapses that lead to significant dendritic spines loss.^121^ In hippocampal neuronal culture, persistent addition of soluble Aβ‐derived oligomers resulted in the thinning of spines and reductions in spinal density. This structural deterioration was concurrent with decreases in cytoskeletal protein drebrin.^122^ In a triple transgenic animal model of AD, dendritic spine density was reduced not only near Aβ plaque sites but also in distal areas that accumulate both soluble Aβ and hyperphosphorylated tau.^123^, ^124^, ^125^ These reports substantiate a critical role of Aβ in altering the synaptic microstructure.

## 5 GLUA1 AND POST‐TRANSLATIONAL MODIFICATIONS

Post‐translational modifications of the GluA1 subunit can alter the performance of the subunit‐containing receptor. This is achieved by altering the binding properties of the subunit, the recruitment of regulatory accessory proteins and complexes, and the overall probability of synaptic plasticity. One of the most common PTMs occurring in neurons is phosphorylation, which can regulate each of the four AMPAR subunits. Phosphorylation is critical for synaptic plasticity, with LTD induction generally associated with dephosphorylation of major PKA sites while LTP induction is associated with dephosphorylation of CaMKII sites.^126^ Experiments with phosphomimetic knock‐in mice have demonstrated that S831 and S845 are critical phosphorylation sites on the GluA1 subunit. For example, phosphorylation of GluA1 S831 increases channel conductance^81^, ^127^ while phosphorylation of S845 increases single‐channel open probability.^94^ GluA1 phosphorylation has also been shown to alter the spiking patterns of CA1 cells in vivo via enhanced AMPAR‐evoked spiking.^128^ Notably, these sites exhibit lowered thresholds for LTP in response to weak, theta‐burst stimulation increasing the probability of synaptic plasticity.^129^ RNAi experiments have further shown that phosphorylation of GluA1 at S845 is required for spatial memory formation.^130^ The kinase PKC can increase GluA1 S818 phosphorylation which in turn recruits the exocytosis‐associated protein 4.1N to maintain LTP.^89^, ^100^ In general, activity‐dependent phosphorylation of GluA1 is associated with AMPAR delivery to the synapse, while dephosphorylation is associated with AMPAR endocytosis and synaptic weakening^131^ (the role of PTMs in GluA1 distribution are covered in the section ‘functional properties of GluA1’). Studies in rat hippocampus have further indicated that activation of different NMDA subpopulations greatly influences GluA1 phosphorylation, suggesting a mechanism for NMDAR‐dependent synaptic plasticity.

α‐amino‐3‐hydroxy‐5‐methyl‐4‐isoxazolepropionic acid receptors are susceptible to lysine acetylation at the C‐terminal, reducing AMPAR internalization and degradation, leading to increased cell surface localization and stabilization.^53^ On the other hand, ubiquitination by E3 ligases, including Nedd4, facilitates AMPAR internalization and degradation.^53^ Finally, S‐nitrosylation of GluA1 at S831 has been shown to facilitate AMPAR conductance and endocytosis via AP2 binding in HEK293 cells overexpressing GluA1.^132^ Diering and Huganir eloquently lay out all 11 known PTMs occurring on GluA1, including seven phosphorylations, two palmitoylations, one S‐nitrosylation and one ubiquitination, each of which can uniquely modify the subunit to exhibit different properties.^27^

Due to the influence of PTMs on AMPAR insertion, trafficking and stability, they have been investigated in the aetiology of AD. To date, one class of post‐translational modification has stood out in the body of AD research. AMPAR ubiquitination and subsequent removal of AMPARs from the plasma membrane has been demonstrated in cultured neurons exposed to soluble Aβ oligomers. This exposure produced reductions in AMPAR currents as well as spine loss.^133^ More recently, Aβ exposure was shown to increase GluA1 ubiquitination (particularly at lysine 63) concurrent with increased AMPAR degradation in cortical neuron culture and AD brain lysates. This was observed to occur in tandem with increases in the E3 ligase Nedd4, while the expression of deubiquitinating enzymes was decreased.^134^

## 6 GLUA1 AND SYNAPTIC PROTEIN INTERAACTIONS

GluA1 properties are heavily regulated by synaptic protein interactions. For example, the cytoskeletal protein Arc acts as an immediate early gene that can be induced in the nucleus in response to excitatory activity.^135^ In the nucleus, Arc regulates GluA1 transcription to modulate synaptic strength. Arc specifically decreases GluA1 transcription by regulating endocytosis of AMPAR.^136^, ^137^ CaMKII‐mediated phosphorylation of the TARP stargazing promotes binding to PSD‐95, subsequently promoting retention of GluA1‐containing AMPARs to the synapse. If GluA1 binds SAP97 at the PDZ domain following CaMKII activation, GluA1‐containing receptors are recruited to the synapse via binding of the motor protein myosin VI.^138^ Finally, binding of the PDZ domain with nexin27 promotes the maintenance of basal AMPAR surface expression and in mediating AMPAR insertion during LTP.^139^ An interesting feedback loop was found wherein retinoic acid (a regulator of GluA1 protein synthesis) is triggered by silencing of synaptic transmission, consequently stimulating GluA1 in the synapse.^140^

Pathologically, Aβ has been shown to alter the synaptic distribution of the kinase CaMKII. For instance, in APP transgenic mice, the pool of CaMKII is reduced and in cortical neurons treated with Aβ oligomer, decreased CaMKII clusters are present at the synapse.^57^ The restoration of CaMKII and AMPAR‐mediated transmission further implicates Aβ as a regulator of the subcellular distribution of CaMKII and destabilizer of synaptic AMPARs and synaptic potentiation. On the other hand, animal models of tauopathy have shown through sophisticated imaging strategies that although the synaptic density of AMPARs, synaptic proteins like PSD‐95, GluN1 and GluA1 are reduced.^141^ These findings suggest that synapses exposed to aberrant tau may exert ultrastructural changes through the alteration of synaptic proteins which can significantly alter synaptic function in pathological states like AD.

## 7 CONCLUSIONS AND FUTURE PERSPECTIVES

In the United States alone, AD impacts over 5 million and is estimated to bear a 200 billion dollar per year economic burden. The disease is particularly pressing as society faces an increased ageing population. Thus, elucidating the underlying mechanisms of cognitive and memory function and decline is especially important. An increasing body of evidence has implicated Aβ and tau aggregates to a host of deleterious neurobiological processes such as reductions in excitatory synaptic transmission, loss of dendritic spines and excitotoxic neuronal death. Indeed, a hallmark of AD aetiology includes dysregulated synaptic transmission that has been largely attributed to dendritic spine loss, deficits in glutamatergic synaptic transmission and impaired cognition, learning and memory capacity. Interestingly, these phenomena have been found to occur prior to the detection of pathological plaques and neuronal loss. This beckons the question of whether any of the above described molecular pathologies are consequence or cause. Regardless, the evidence is quite clear in that AD affected subjects distinctly suffer inhibited LTP due to reductions in excitatory neurotransmission. As a major excitatory receptor in the central nervous system, AMPARs have been a focus of AD research. Indeed, as previously discussed, AMPAR abundance, trafficking, localization and function have all been shown to be dysregulated across various AD models. The receptor subunit GluA1 is perhaps the most well‐studied component among AMPARs. Through a variety of molecular mechanisms including trafficking, recycling, modifications of microstructural properties, PTMs and modulations due to interactions with critical synaptic proteins, GluA1‐containing AMPARs are a medium for the aetiology of AD and similar neurogenerative pathologies.

In this review, we discussed various aspects of GluA1’s role in establishing synaptic plasticity, a fundamental component of cognitive processes like learning and memory. Specifically, we highlighted peer‐reviewed findings in the areas of GluA1 trafficking to and from the synapse, microstructural adaptations caused by GluA1‐containing receptor modulation, single‐channel functional modulation and well‐studied PTMs that influence interaction of the subunit with regulatory proteins. Altogether, these variations provide ample possibilities for GluA1‐containing AMPARs to modify synaptic transmission to shape LTD/LTP and subsequently learning and memory. Many questions stand including the resolution of a complex network of subunit diversity, PTMs and regulatory protein interactions. Additionally, potential crosstalk between PTM‐conferring proteins, compensatory mechanisms and feedback loops remain to be fully characterized. Undoubtedly, the list of proteins able to regulate synaptic AMPAR levels and their activity remains incomplete. Further, it is likely that live, high‐resolution microscopy has only begun to reveal the intricacies of molecular movement at the synapse. Finally, pathology‐driven changes in GluA1 dynamics and novel therapeutic strategies to address these remain to undergo a battery of clinical investigation.

## CONFLICT OF INTEREST

The authors declare that they have no competing interests.

## AUTHOR CONTRIBUTIONS

WQ, BY and QL conceptualized and design the manuscript. WQ, JL, RC and XZ searched the literature and wrote the manuscript. WY and BL viewed, edited and approved the manuscript. All authors read and approved the final manuscript.

## Data Availability

Data sharing is not applicable to this article, so no new data were created or analysed in this study.

## References

[1] Hu N‐W , Ondrejcak T , Rowan MJ . Glutamate receptors in preclinical research on Alzheimer's disease: update on recent advances. Pharmacol Biochem Behav. 2012;100(4):855‐862. 10.1016/j.pbb.2011.04.013 21536064

[2] Forner S , Baglietto‐Vargas D , Martini AC , Trujillo‐Estrada L , LaFerla FM . Synaptic impairment in Alzheimer’s disease: a dysregulated symphony. Trends Neurosci. 2017;40(6):347‐357. 10.1016/j.tins.2017.04.002 28494972

[3] Styr B , Slutsky I . Imbalance between firing homeostasis and synaptic plasticity drives early‐phase Alzheimer’s disease. Nat Neurosci. 2018;21(4):463‐473. 10.1038/s41593-018-0080-x 29403035 PMC6533171

[4] Chakroborty S , Hill ES , Christian DT , et al. Reduced presynaptic vesicle stores mediate cellular and network plasticity defects in an early‐stage mouse model of Alzheimer’s disease. Mol Neurodegener. 2019;14(1):7 10.1186/s13024-019-0307-7 30670054 PMC6343260

[5] Guntupalli S , Widagdo J , Anggono V . Amyloid‐beta‐induced dysregulation of AMPA receptor trafficking. Neural Plast. 2016;2016:3204519 10.1155/2016/3204519 27073700 PMC4814684

[6] Nieweg K , Andreyeva A , van Stegen B , Tanriöver G , Gottmann K . Alzheimer's disease‐related amyloid‐β induces synaptotoxicity in human iPS cell‐derived neurons. Cell Death Dis. 2015;6(4):e1709 10.1038/cddis.2015.72 25837485 PMC4650541

[7] Sheng M , Sabatini BL , Südhof TC . Synapses and Alzheimer’s disease. Cold Spring Harb Perspect Biol. 2012; 4(5):a005777 10.1101/cshperspect.a005777. 22491782 PMC3331702

[8] Henley JM , Wilkinson KA . Synaptic AMPA receptor composition in development, plasticity and disease. Nat Rev Neurosci. 2016;17(6):337‐350. 10.1038/nrn.2016.37 27080385

[9] Cheignon C , Tomas M , Bonnefont‐Rousselot D , Faller P , Hureau C , Collin F . Oxidative stress and the amyloid beta peptide in Alzheimer’s disease. Redox Biol. 2018;14:450‐464. 10.1016/j.redox.2017.10.014 29080524 PMC5680523

[10] Mroczko B , Groblewska M , Litman‐Zawadzka A , Kornhuber J , Lewczuk P . Amyloid β oligomers (AβOs) in Alzheimer’s disease. J Neural Transm. 2018;125(2):177‐191. 10.1007/s00702-017-1820-x 29196815

[11] Morrone CD , Bazzigaluppi P , Beckett TL , et al. Regional differences in Alzheimer’s disease pathology confound behavioural rescue after amyloid‐β attenuation. Brain. 2019;143(1):359‐373. 10.1093/brain/awz371 PMC6935751 31782760

[12] Ahmed T , Zahid S , Mahboob A , Mehpara FS . Cholinergic system and post‐translational modifications: an insight on the role in Alzheimer's disease. Curr Neuropharmacol. 2017;15(4):480‐494. 10.2174/1570159X14666160325121145 27012953 PMC5543671

[13] Marcelli S , Corbo M , Iannuzzi F , et al. The involvement of post‐translational modifications in Alzheimer's disease. Curr Alzheimer Res. 2018;15(4):313‐335. 10.2174/1567205014666170505095109 28474569

[14] Tsatsanis A , Dickens S , Kwok JCF , Wong BX , Duce JA . Post translational modulation of β‐amyloid precursor protein trafficking to the cell surface alters neuronal iron homeostasis. Neurochem Res. 2019;44(6):1367‐1374. 10.1007/s11064-019-02747-y 30796750 PMC6525264

[15] Holtmaat A , Svoboda K . Experience‐dependent structural synaptic plasticity in the mammalian brain. Nat Rev Neurosci. 2009;10(9):647 10.1038/nrn2699 19693029

[16] Lu W , Shi Y , Jackson AC , et al. Subunit composition of synaptic AMPA receptors revealed by a single‐cell genetic approach. Neuron. 2009;62(2):254‐268. 10.1016/j.neuron.2009.02.027 19409270 PMC3632349

[17] Terashima A , Suh YH , Isaac JTR . The AMPA receptor subunit GluA1 is required for CA1 hippocampal long‐term potentiation but is not essential for synaptic transmission. Neurochem Res. 2019;44(3):549‐561. 10.1007/s11064-017-2425-3 29098531

[18] Huganir Richard L , Nicoll RA . AMPARs and synaptic plasticity: the last 25 years. Neuron. 2013;80(3):704‐717. 10.1016/j.neuron.2013.10.025 24183021 PMC4195488

[19] Pozo K , Goda Y . Unraveling mechanisms of homeostatic synaptic plasticity. Neuron. 2010;66(3):337‐351. 10.1016/j.neuron.2010.04.028 20471348 PMC3021747

[20] Lohmann C , Kessels HW . The developmental stages of synaptic plasticity. J Physiol. 2014;592(1):13‐31. 10.1113/jphysiol.2012.235119 24144877 PMC3903349

[21] Shi SH . Amersham Biosciences & Science Prize. AMPA receptor dynamics and synaptic plasticity. Science. 2001;294(5548):1851 10.1126/science.1067844 11729297

[22] Hayashi Y , Shi SH , Esteban JA , Piccini A , Poncer JC , Malinow R . Driving AMPA receptors into synapses by LTP and CaMKII: requirement for GluR1 and PDZ domain interaction. Science. 2000;287(5461):2262 10.1126/science.287.5461.2262. 10731148

[23] Granger AJ , Shi Y , Lu W , Cerpas M , Nicoll RA . LTP requires a reserve pool of glutamate receptors independent of subunit type. Nature. 2013;493(7433):495‐500. 10.1038/nature11775 23235828 PMC3998843

[24] Hausser A , Schlett K . Coordination of AMPA receptor trafficking by Rab GTPases. Small GTPases. 2019;10(6):419‐432. 10.1080/21541248.2017.1337546 28628388 PMC6748377

[25] Buonarati OR , Hammes EA , Watson JF , Greger IH , Hell JW . Mechanisms of postsynaptic localization of AMPA‐type glutamate receptors and their regulation during long‐term potentiation. Sci Signal. 2019;12(562):eaar6889 10.1126/scisignal.aar6889 30600260 PMC7175813

[26] Lussier MP , Sanz‐Clemente A , Roche KW . Dynamic regulation of NMDA and AMPA receptors by posttranslational modifications. J Biol Chem. 2015;290(48):28596‐28603. 10.1074/jbc.R115.652750 26453298 PMC4661374

[27] Diering GH , Huganir RL . The AMPA receptor code of synaptic plasticity. Neuron. 2018;100(2):314‐329. 10.1016/j.neuron.2018.10.018 30359599 PMC6214363

[28] Park M . AMPA Receptor trafficking for postsynaptic potentiation. Review. Front Cell Neurosci. 2018;12:361 10.3389/fncel.2018.00361 30364291 PMC6193507

[29] Ho VM , Lee JA , Martin KC . The cell biology of synaptic plasticity. Science. 2011;334(6056):623‐628. 10.1126/science.1209236 22053042 PMC3286636

[30] Androuin A , Potier B , Nägerl UV , et al. Evidence for altered dendritic spine compartmentalization in Alzheimer’s disease and functional effects in a mouse model. Acta Neuropathol. 2018;135(6):839‐854. 10.1007/s00401-018-1847-6 29696365

[31] Boros BD , Greathouse KM , Gearing M , Herskowitz JH . Dendritic spine remodeling accompanies Alzheimer's disease pathology and genetic susceptibility in cognitively normal aging. Neurobiol Aging. 2019;73:92‐103. 10.1016/j.neurobiolaging.2018.09.003 30339964 PMC6251733

[32] Skaper SD , Facci L , Zusso M , Giusti P . Synaptic plasticity, dementia and Alzheimer disease. CNS Neurol Disorder. 2017;16(3):220‐233. 10.2174/1871527316666170113120853 28088900

[33] Aarsland D , Creese B , Politis M , et al. Cognitive decline in Parkinson disease. Nat Rev Neurol. 2017;13(4):217‐231. 10.1038/nrneurol.2017.27 28257128 PMC5643027

[34] Osimo EF , Beck K , Reis Marques T , Howes OD . Synaptic loss in schizophrenia: a meta‐analysis and systematic review of synaptic protein and mRNA measures. Mol Psychiatry. 2019;24(4):549‐561. 10.1038/s41380-018-0041-5 29511299 PMC6004314

[35] Fukata Y , Fukata M . Epilepsy and synaptic proteins. Curr Opin Neurobiol. 2017;45:1‐8. 10.1016/j.conb.2017.02.001 28219682

[36] Roelfsema PR , Holtmaat A . Control of synaptic plasticity in deep cortical networks. Nat Rev Neurosci. 2018;19(3):166‐180. 10.1038/nrn.2018.6 29449713

[37] Humeau Y , Choquet D . The next generation of approaches to investigate the link between synaptic plasticity and learning. Nat Neurosci. 2019;22(10):1536‐1543. 10.1038/s41593-019-0480-6 31477899

[38] Almeida CG , Tampellini D , Takahashi RH , et al. Beta‐amyloid accumulation in APP mutant neurons reduces PSD‐95 and GluR1 in synapses. Neurobiology of disease. 2005;20(2):187 10.1016/j.nbd.2005.02.008 16242627

[39] Yang Y , Kim J , Kim HY , et al. Amyloid‐beta oligomers may impair SNARE‐mediated exocytosis by direct binding to syntaxin 1a. Cell Rep. 2015;12(8):1244 10.1016/j.celrep.2015.07.044. 26279571 PMC4955600

[40] Deak F . Neuronal vesicular trafficking and release in age‐related cognitive impairment. J Gerontol A Biol Sci Med Sci. 2014;69(11):1325 10.1093/gerona/glu061 24809352

[41] Cao J , Hou J , Ping J , Cai D . Advances in developing novel therapeutic strategies for Alzheimer’s disease. Mol Neurodegener. 2018;13(1):64 10.1186/s13024-018-0299-8 30541602 PMC6291983

[42] Henstridge CM , Pickett E , Spires‐Jones TL . Synaptic pathology: a shared mechanism in neurological disease. Ageing Res Rev. 2016;28:72‐84. 10.1016/j.arr.2016.04.005 27108053

[43] Sevigny J , Chiao P , Bussière T , et al. The antibody aducanumab reduces Aβ plaques in Alzheimer’s disease. Nature. 2016;537(7618):50‐56. 10.1038/nature19323 27582220

[44] Takahashi RH , Nagao T , Gouras GK . Plaque formation and the intraneuronal accumulation of β‐amyloid in Alzheimer's disease. Pathol Int. 2017;67(4):185‐193. 10.1111/pin.12520 28261941

[45] Guo C , Jeong H‐H , Hsieh Y‐C , et al. Tau activates transposable elements in Alzheimer’s disease. Cell Rep. 2018;23(10):2874‐2880. 10.1016/j.celrep.2018.05.004 29874575 PMC6181645

[46] Crimins JL , Pooler A , Polydoro M , Luebke JI , Spires‐Jones TL . The intersection of amyloid beta and tau in glutamatergic synaptic dysfunction and collapse in Alzheimer's disease. Ageing Res Rev. 2013;12(3):757‐763. 10.1016/j.arr.2013.03.002 23528367 PMC3735866

[47] Shankar GM , Li S , Mehta TH , et al. Amyloid‐beta protein dimers isolated directly from Alzheimer's brains impair synaptic plasticity and memory. NatMed. 2008;14(8):837 10.1038/nm1782 PMC2772133 18568035

[48] Guntupalli S , Jang SE , Zhu T , Huganir RL , Widagdo J , Anggono V . GluA1 subunit ubiquitination mediates amyloid‐beta‐induced loss of surface alpha‐amino‐3‐hydroxy‐5‐methyl‐4‐isoxazolepropionic acid (AMPA) receptors. J Biol Chem. 2017;292(20):8186 10.1074/jbc.M116.774554 28377502 PMC5437227

[49] Tanaka H , Sakaguchi D , Hirano T . Amyloid‐β oligomers suppress subunit‐specific glutamate receptor increase during LTP. Alzheimers Demen. 2019;5:797‐808. 10.1016/j.trci.2019.10.003 PMC6880111 31788535

[50] Duman RS , Shinohara R , Fogaça MV , Hare B . Neurobiology of rapid‐acting antidepressants: convergent effects on GluA1‐synaptic function. Mol Psychiatry. 2019;24(12):1816‐1832. 10.1038/s41380-019-0400-x 30894661 PMC6754322

[51] Hu N‐W , Corbett GT , Moore S , et al. Extracellular forms of Aβ and Tau from iPSC models of Alzheimer’s disease disrupt synaptic plasticity. Cell Rep. 2018;23(7):1932‐1938. 10.1016/j.celrep.2018.04.040 29768194 PMC5972225

[52] Middei S , Roberto A , Berretta N , et al. Learning discloses abnormal structural and functional plasticity at hippocampal synapses in the APP23 mouse model of Alzheimer's disease. Learn Mem. 2010;17(5):236‐240. 10.1101/lm.1748310 20404004

[53] Walsh DM , Selkoe DJ . A beta oligomers ‐ a decade of discovery. J Neuro Chem. 2007;101(5):1172. 10.1111/j.1471-4159.2006.04426.x 17286590

[54] Reiss Allison B , Arain Hirra A , Stecker Mark M , Siegart Nicolle M , Kasselman LJ . Amyloid toxicity in Alzheimer’s disease. Rev Neurosci. 2018;613‐627. 10.1515/revneuro-2017-0063 29447116

[55] Zhao WQ , Santini F , Breese R , et al. Inhibition of calcineurin‐mediated endocytosis and alpha‐amino‐3‐hydroxy‐5‐methyl‐4‐isoxazolepropionic acid (AMPA) receptors prevents amyloid beta oligomer‐induced synaptic disruption. J Biol Chem. 2010;285(10):7619 10.1074/jbc.M109.057182 20032460 PMC2844209

[56] Whitcomb DJ , Hogg EL , Regan P , et al. Intracellular oligomeric amyloid‐beta rapidly regulates GluA1 subunit of AMPA receptor in the hippocampus. Sci Rep. 2015;5(1):10934 10.1038/srep10934. 26055072 PMC4460729

[57] Gu Z , Liu W , Yan Z . {beta}‐Amyloid impairs AMPA receptor trafficking and function by reducing Ca2+/calmodulin‐dependent protein kinase II synaptic distribution. J Biol Chem. 2009;284(16):10639 10.1074/jbc.M806508200. 19240035 PMC2667751

[58] Schurmann B , Bermingham DP , Kopeikina KJ , et al. A novel role for the late‐onset Alzheimer's disease (LOAD)‐associated protein Bin1 in regulating postsynaptic trafficking and glutamatergic signaling. Mol Psychiatry 2020;25(9):2000‐2016. 10.1038/s41380-019-0407-3 30967682 PMC6785379

[59] Baglietto‐Vargas D , Prieto GA , Limon A , et al. Impaired AMPA signaling and cytoskeletal alterations induce early synaptic dysfunction in a mouse model of Alzheimer's disease. Aging Cell. 2018;17(4):e12791 10.1111/acel.12791 29877034 PMC6052400

[60] Schwarz LA , Hall BJ , Patrick GN . Activity‐dependent ubiquitination of GluA1 mediates a distinct AMPA receptor endocytosis and sorting pathway. J Neurosci. 2010;30(49):16718‐16729. 10.1523/JNEUROSCI.3686-10.2010 21148011 PMC3079366

[61] Eh C , Mj S , Dg F , et al. AMPA receptor downscaling at the onset of Alzheimer's disease pathology in double knockin mice. Proc Natl Acad Sci USA. 2006;103(9):3410‐3415. 10.1073/pnas.0507313103 16492745 PMC1413872

[62] Gilbert J , Shu S , Yang X , Lu Y , Zhu LQ , Man HY . β‐Amyloid triggers aberrant over‐scaling of homeostatic synaptic plasticity. Acta Neuropathologica Communications. 2016;4(1):131 10.1186/s40478-016-0398-0 27955702 PMC5154098

[63] Kim S , Violette CJ , Ziff EB . Reduction of increased calcineurin activity rescues impaired homeostatic synaptic plasticity in presenilin 1 M146V mutant. Neurobiol Aging. 2015;36(12):3239‐3246. 10.1016/j.neurobiolaging.2015.09.007 26455952 PMC4641803

[64] Ss J , Hj C . Emerging link between Alzheimer's disease and homeostatic synaptic plasticity. Neural Plast. 2016;2016:7969272 10.1155/2016/7969272 27019755 PMC4785275

[65] Arnold SE , Arvanitakis Z , Macauley‐Rambach SL , et al. Brain insulin resistance in type 2 diabetes and Alzheimer disease: concepts and conundrums. Nat Rev Neurol. 2018;14(3):168‐181. 10.1038/nrneurol.2017.185 29377010 PMC6098968

[66] Willette AA , Bendlin BB , Starks EJ , et al. Association of insulin resistance with cerebral glucose uptake in late middle‐aged adults at risk for Alzheimer disease. JAMA Neurology. 2015;72(9):1013‐1020. 10.1001/jamaneurol.2015.0613 26214150 PMC4570876

[67] Ferreira LSS , Fernandes CS , Vieira MNN , De Felice FG . Insulin resistance in Alzheimer's disease. Mini Review. Front Neurosci. 2018;12:830 10.3389/fnins.2018.00830 30542257 PMC6277874

[68] Grillo CA , Piroli GG , Lawrence RC , et al. Hippocampal insulin resistance impairs spatial learning and synaptic plasticity. Diabetes. 2015;64(11):3927 10.2337/db15-0596 26216852 PMC4613975

[69] Spinelli M , Fusco S , Mainardi M , et al. Brain insulin resistance impairs hippocampal synaptic plasticity and memory by increasing GluA1 palmitoylation through FoxO3a. Nat Commun. 2017;8(1):1‐14. 10.1038/s41467-017-02221-9. 29222408 PMC5722929

[70] Olivito L , Saccone P , Perri V , et al. Phosphorylation of the AMPA receptor GluA1 subunit regulates memory load capacity. Brain Struct Funct. 2016;221(1):591‐603. 10.1007/s00429-014-0927-1 25381005 PMC4425615

[71] Zhao LX , Chen MW , Qian Y , et al. M1 Muscarinic receptor activation rescues beta‐amyloid‐induced cognitive impairment through AMPA Receptor GluA1 subunit. Neuroscience. 2019;408:239. 30981860 10.1016/j.neuroscience.2019.04.007

[72] Zhang J , Yin Y , Ji Z , et al. Endophilin2 interacts with GluA1 to mediate AMPA receptor endocytosis induced by oligomeric amyloid‐beta. Neural Plast. 2017;2017:8197085 10.1155/2017/8197085 28758034 PMC5516760

[73] Gao L , Tian M , Zhao HY , et al. TrkB activation by 7, 8‐dihydroxyflavone increases synapse AMPA subunits and ameliorates spatial memory deficits in a mouse model of Alzheimer's disease. JNeurochem. 2016;136(3):620 10.1111/jnc.13432 26577931

[74] Brunetti G , Di Rosa G , Scuto M , et al. Healthspan Maintenance and Prevention of Parkinson’s‐like Phenotypes with Hydroxytyrosol and Oleuropein Aglycone in C. elegans. Int J Mol Sci 2020;21(7):2588 10.3390/ijms21072588 PMC7178172 32276415

[75] Di Rosa G , Brunetti G , Scuto M , et al. Healthspan enhancement by olive polyphenols in *C. elegans* wild type and Parkinson’s models. Int J Mol Sci. 2020;21(11):3893 10.3390/ijms21113893 PMC7312680 32486023

[76] Calabrese V , Cornelius C , Dinkova‐Kostova AT , Calabrese EJ , Mattson MP . Cellular stress responses, the hormesis paradigm, and vitagenes: novel targets for therapeutic intervention in neurodegenerative disorders. Antioxid Redox Signal. 2010;13(11):1763‐1811. 10.1089/ars.2009.3074 20446769 PMC2966482

[77] Fusco R , Cordaro M , Genovese T , et al. Adelmidrol: a new promising antioxidant and anti‐inflammatory therapeutic tool in pulmonary fibrosis. Antioxidants. 2020;9(7):601 10.3390/antiox9070601 PMC7402091 32660140

[78] Scuto M , Di Mauro P , Ontario ML , et al. Nutritional mushroom treatment in meniere’s disease with coriolus versicolor:a rationale for therapeutic intervention in neuroinflammation and antineurodegeneration. Int J Mol Sci. 2020;21(1):284 10.3390/ijms21010284 PMC6981469 31906226

[79] Amara I , Scuto M , Zappalà A , et al. Hericium erinaceus prevents DEHP‐induced mitochondrial dysfunction and apoptosis in PC12 Cells. Int J Mol Sci. 2020;21(6):2138 10.3390/ijms21062138 PMC7139838 32244920

[80] Whitehead G , Regan P , Whitcomb DJ , Cho K . Ca2+‐permeable AMPA receptor: A new perspective on amyloid‐beta mediated pathophysiology of Alzheimer's disease. Neuropharmacology. 2017;112:221‐227. 10.1016/j.neuropharm.2016.08.022 27561971

[81] Derkach V , Barria A , Soderling TR . Ca2+/calmodulin‐kinase II enhances channel conductance of alpha‐amino‐3‐hydroxy‐5‐methyl‐4‐isoxazolepropionate type glutamate receptors. Proc Natil Acad Sci USA. 16 1999;96(6):3269 10.1073/pnas.96.6.3269. PMC15931 10077673

[82] Derkach VA , Oh MC , Guire ES , Soderling TR . Regulatory mechanisms of AMPA receptors in synaptic plasticity. Nat Rev Neurosci. 2007;8(2):101 10.1038/nrn2055 17237803

[83] Sanderson DJ , Good MA , Skelton K , et al. Enhanced long‐term and impaired short‐term spatial memory in GluA1 AMPA receptor subunit knockout mice: Evidence for a dual‐process memory model. Learn Mem. 2009;16(6):379‐386. 10.1101/lm.1339109. 19470654 PMC2704103

[84] Passafaro M , Piech V , Sheng M . Subunit‐specific temporal and spatial patterns of AMPA receptor exocytosis in hippocampal neurons. Nat Neurosci. 2001;4(9):917 10.1038/nn0901-917 11528423

[85] Sanderson TM , Bradley CA , Georgiou J , et al. The probability of neurotransmitter release governs AMPA receptor trafficking via activity‐dependent regulation of mGluR1 surface expression. Cell Rep. 2018;25(13):3631‐3646.e3. 10.1016/j.celrep.2018.12.010 30590038 PMC6315206

[86] Sheng N , Bemben MA , Díaz‐Alonso J , Tao W , Shi YS , Nicoll RA . LTP requires postsynaptic PDZ‐domain interactions with glutamate receptor/auxiliary protein complexes. Proc Natl Acad Sci. 2018;115(15):3948‐3953. 10.1073/pnas.1800719115 29581259 PMC5899490

[87] Wu D , Bacaj T , Morishita W , et al. Postsynaptic synaptotagmins mediate AMPA receptor exocytosis during LTP. Nature. 2017;544(7650):316‐321. 10.1038/nature21720 28355182 PMC5734942

[88] Citri A , Malenka RC . Synaptic plasticity: multiple forms, functions, and mechanisms. Neuropsychopharmacology. 2008;33(1):18 10.1038/sj.npp.1301559 17728696

[89] Lin DT , Makino Y , Sharma K , et al. Regulation of AMPA receptor extrasynaptic insertion by 4.1N, phosphorylation and palmitoylation. Nat Neurosci. 2009;12(7):879‐887. 10.1038/nn.2351. 19503082 PMC2712131

[90] Anggono V , Huganir RL . Regulation of AMPA receptor trafficking and synaptic plasticity. Cur rOpin Neurobiol. 2012;22(3):461 10.1016/j.conb.2011.12.006 PMC3392447 22217700

[91] Mammen AL , Kameyama K , Roche KW , Huganir RL . Phosphorylation of the alpha‐amino‐3‐hydroxy‐5‐methylisoxazole4‐propionic acid receptor GluR1 subunit by calcium/calmodulin‐dependent kinase II. J Biol Chem. 1997;272(51):32528 10.1074/jbc.272.51.32528. 9405465

[92] Kristensen AS , Jenkins MA , Banke TG , et al. Mechanism of Ca2+/calmodulin‐dependent kinase II regulation of AMPA receptor gating. Nat Neurosci. 2011;14(6):727 10.1038/nn.2804 21516102 PMC3102786

[93] Arendt KL , Zhang Z , Ganesan S , et al. Calcineurin mediates homeostatic synaptic plasticity by regulating retinoic acid synthesis. Proc Natl Acad Sci. 2015;112(42):E5744‐E5752. 10.1073/pnas.1510239112 26443861 PMC4620864

[94] Banke TG , Bowie D , Lee H , Huganir RL , Schousboe A , Traynelis SF . Control of GluR1 AMPA receptor function by cAMP‐dependent protein kinase. J Neurosci. 2000;20(1):89‐102. 10.1523/JNEUROSCI.20-01-00089.2000 10627585 PMC6774102

[95] Oh MC , Derkach VA , Guire ES , Soderling TR . Extrasynaptic membrane trafficking regulated by GluR1 Serine 845 phosphorylation primes AMPA receptors for long‐term potentiation. J Biol Chem. 2006;281(2):752‐758. 10.1074/jbc.M509677200 16272153

[96] Man HY , Sekine‐Aizawa Y , Huganir RL . Regulation of {alpha}‐amino‐3‐hydroxy‐5‐methyl‐4‐isoxazolepropionic acid receptor trafficking through PKA phosphorylation of the Glu receptor 1 subunit. Proc Natl Acad Sci USA. 2007;104(9):3579. 17360685 10.1073/pnas.0611698104 PMC1805611

[97] Lee HK , Takamiya K , Han JS , et al. Phosphorylation of the AMPA Receptor GluR1 subunit is required for synaptic plasticity and retention of spatial memory. Cell. 2003;112(5):631‐643. 10.1016/S0092-8674(03)00122-3 12628184

[98] Lee HK , Takamiya K , He K , Song L , Huganir RL . Specific roles of AMPA receptor subunit GluR1 (GluA1) phosphorylation sites in regulating synaptic plasticity in the CA1 region of hippocampus. J Neurophysiol. 2010;103(1):479 10.1152/jn.00835.2009 19906877 PMC2807233

[99] Ehlers MD . Reinsertion or degradation of AMPA receptors determined by activity‐dependent endocytic sorting. Neuron. 2000;28(2):511 10.1016/S0896-6273(00)00129-X 11144360

[100] Boehm J , Ehrlich I , Hsieh H , Malinow R . Two mutations preventing PDZ‐protein interactions of GluR1 have opposite effects on synaptic plasticity. LearnMem. 2006;13(5):562 10.1101/lm.253506 16980545

[101] Sanderson Jennifer L , Gorski Jessica A , Dell’Acqua ML . NMDA receptor‐dependent LTD requires transient synaptic incorporation of Ca2+‐Permeable AMPARs Mediated by AKAP150‐Anchored PKA and calcineurin. Neuron. 2016;89(5):1000‐1015. 10.1016/j.neuron.2016.01.043 26938443 PMC4914360

[102] Hangen E , Cordelières FP , Petersen JD , Choquet D , Coussen F . Neuronal activity and intracellular calcium levels regulate intracellular transport of newly synthesized AMPAR. Cell Rep. 2018;24(4):1001‐1012.e3. 10.1016/j.celrep.2018.06.095 30044968 PMC6083039

[103] Hasegawa S , Fukushima H , Hosoda H , et al. Hippocampal clock regulates memory retrieval via Dopamine and PKA‐induced GluA1 phosphorylation. Nat Commun. 2019;10(1):5766 10.1038/s41467-019-13554y 31852900 PMC6920429

[104] Sanderson JL , Gorski JA , Dell'Acqua ML . NMDA receptor‐dependent LTD requires transient synaptic incorporation of Ca(2)(+)‐permeable AMPARs mediated by AKAP150‐anchored PKA and calcineurin. Neuron. 2016; 89(5):1000‐1015. 10.1016/j.neuron.2016.01.043 26938443 10.1016/j.neuron.2016.01.043 PMC4914360

[105] Lee HK , Kameyama K , Huganir RL , Bear MF . NMDA induces long‐term synaptic depression and dephosphorylation of the GluR1 subunit of AMPA receptors in hippocampus. Neuron. 1998;21(5):1151 10.1016/S0896-6273(00)80632-7. 9856470

[106] Chater TE , Goda Y . The role of AMPA receptors in postsynaptic mechanisms of synaptic plasticity. Front Cell Neurosci. 2014;8: 10.3389/fncel.2014.00401 PMC4245900 25505875

[107] Ehrlich I , Malinow R . Postsynaptic density 95 controls AMPA receptor incorporation during long‐term potentiation and experience‐driven synaptic plasticity. J Neurosci. 2004;24(4):916‐927. 10.1523/JNEUROSCI.4733-03.2004 14749436 PMC6729816

[108] Opazo P , Choquet D . A three‐step model for the synaptic recruitment of AMPA receptors. MolCellNeurosci. 2011;46(1):1 10.1016/j.mcn.2010.08.014 20817097

[109] Lu W , Isozaki K , Roche KW , Nicoll RA . Synaptic targeting of AMPA receptors is regulated by a CaMKII site in the first intracellular loop of GluA1. Proceedings of the National Academy of Sciences. 2010;107(51):22266‐22271. 10.1073/pnas.1016289107. PMC3009812 21135237

[110] Park M , Penick EC , Edwards JG , Kauer JA , Ehlers MD . Recycling endosomes supply AMPA receptors for LTP. Science. 2004;305(5692):1972‐1975. 10.1126/science.1102026 15448273

[111] Patterson MA , Szatmari EM , Yasuda R . AMPA receptors are exocytosed in stimulated spines and adjacent dendrites in a Ras‐ERK‐dependent manner during long‐term potentiation. Proc Natl Acad Sciences. 2010;107(36):15951‐15956. 10.1073/pnas.0913875107. PMC2936631 20733080

[112] Kennedy MJ , Davison IG , Robinson CG , Ehlers MD . Syntaxin‐4 defines a domain for activity‐dependent exocytosis in dendritic spines. Cell. 2010;141(3):524‐535. 10.1016/j.cell.2010.02.042 20434989 PMC2874581

[113] Hoover BR , Reed MN , Su J , et al. Tau mislocalization to dendritic spines mediates synaptic dysfunction independently of neurodegeneration. Neuron. 2010;68(6):1067‐1081. 10.1016/j.neuron.2010.11.030 21172610 PMC3026458

[114] Zempel H , Thies E , Mandelkow E , Mandelkow EM . Abeta oligomers cause localized Ca(2+) elevation, missorting of endogenous Tau into dendrites, Tau phosphorylation, and destruction of microtubules and spines. J Neurosci. 2010;30(36):11938‐11950. 10.1523/JNEUROSCI.2357-10.2010 20826658 PMC6633549

[115] Polanco JC , Li C , Bodea L‐G , Martinez‐Marmol R , Meunier FA , Götz J . Amyloid‐β and tau complexity — towards improved biomarkers and targeted therapies. Nat Rev Neurol. 2018;14(1):22‐39. 10.1038/nrneurol.2017.162 29242522

[116] Hastings MH , Man H‐Y . Synaptic capture of laterally diffusing AMPA Receptors – an idea that stuck. Trends Neurosci. 2018;41(6):330‐332. 10.1016/j.tins.2018.03.016 29801525

[117] Zhang Y , Cudmore RH , Lin DT , Linden DJ , Huganir RL . Visualization of NMDA receptor‐dependent AMPA receptor synaptic plasticity in vivo. Nat Neurosci. 2015;18(3):402 10.1038/nn.3936 25643295 PMC4339371

[118] Middei S , Spalloni A , Longone P , et al. CREB selectively controls learning‐induced structural remodeling of neurons. Learn Mem. 2012;19(8):330‐336. 10.1101/lm.025817.112. 22815537

[119] Kopec CD , Real E , Kessels HW , Malinow R . Links structural and functional plasticity at excitatory synapses. J Neurosci. 2007;27(50):13706‐13718. 10.1523/JNEUROSCI.3503-07.2007 18077682 PMC6673607

[120] Zhou Z , Liu A , Xia S , et al. The C‐terminal tails of endogenous GluA1 and GluA2 differentially contribute to hippocampal synaptic plasticity and learning. Nat Neurosci. 2018;21:50–62. 29230056 10.1038/s41593-017-0030-z

[121] Brito‐Moreira J , Lourenco MV , Oliveira MM , et al. Interaction of amyloid‐β (Aβ) oligomers with neurexin 2α and neuroligin 1 mediates synapse damage and memory loss in mice. J Biol Chem. 2017;292(18):7327‐7337. 10.1074/jbc.M116.761189 28283575 PMC5418035

[122] Lacor PN , Buniel MC , Furlow PW , et al. Abeta oligomer‐induced aberrations in synapse composition, shape, and density provide a molecular basis for loss of connectivity in Alzheimer's disease. J Neurosci. 2007;27(4):796‐807. 10.1523/JNEUROSCI.3501-06.2007 17251419 PMC6672917

[123] Bittner T , Fuhrmann M , Burgold S , et al. Multiple events lead to dendritic spine loss in triple transgenic Alzheimer's disease mice. PLoS ONE. 2010;5(11):e15477 10.1371/journal.pone.0015477. 21103384 PMC2982845

[124] Ittner A , Ittner LM . Dendritic tau in Alzheimer’s disease. Neuron. 2018;99(1):13‐27. 10.1016/j.neuron.2018.06.003 30001506

[125] Li S , Jin M , Liu L , Dang Y , Ostaszewski BL , Selkoe DJ . Decoding the synaptic dysfunction of bioactive human AD brain soluble Aβ to inspire novel therapeutic avenues for Alzheimer’s disease. Acta Neuropathologica Commun. 2018;6(1):121 10.1186/s40478-018-0626-x PMC6225562 30409172

[126] Lee HK , Barbarosie M , Kameyama K , Bear MF , Huganir RL . Regulation of distinct AMPA receptor phosphorylation sites during bidirectional synaptic plasticity. Nature. 2000;405(6789):955‐959. 10.1038/35016089 10879537

[127] Barria A , Derkach V , Soderling T . Identification of the Ca2+/calmodulin‐dependent protein kinase II regulatory phosphorylation site in the alpha‐amino‐3‐hydroxyl‐5‐methyl‐4‐isoxazole‐propionate‐type glutamate receptor. J Biol Chem. 1997;272(52):32727 10.1074/jbc.272.52.32727. 9407043

[128] Barkoczi B , Juhasz G , Averkin RG , et al. GluA1 phosphorylation alters evoked firing pattern in vivo. Neural Plast. 2012;2012:286215 10.1155/2012/286215 22567428 PMC3337492

[129] Makino Y , Johnson RC , Yu Y , Takamiya K , Huganir RL . Enhanced synaptic plasticity in mice with phosphomimetic mutation of the GluA1 AMPA receptor. Proc Natl Acad Sci. 2011;108(20):8450‐8455. 10.1073/pnas.1105261108. 21536866 PMC3100939

[130] Ferretti V , Perri V , Cristofoli A , et al. Phosphorylation of S845 GluA1 AMPA receptors modulates spatial memory and structural plasticity in the ventral striatum. Brain Struct Funct. 2015;220(5):2653 10.1007/s00429-014-0816-7 24942137

[131] Middei S , Ammassari‐Teule M , Marie H . Synaptic plasticity under learning challenge. Neurobiol Learn Mem. 2014;115:108 10.1016/j.nlm.2014.08.001 25132316

[132] Selvakumar B , Jenkins MA , Hussain NK , Huganir RL , Traynelis SF , Snyder SH . S‐nitrosylation of AMPA receptor GluA1 regulates phosphorylation, single‐channel conductance, and endocytosis. Proceedings of the National Academy of Sciences. 2013;110(3):1077‐1082. 10.1073/pnas.1221295110 PMC3549090 23277581

[133] Rodrigues EM , Scudder SL , Goo MS , Patrick GN . Abeta‐induced synaptic alterations require the E3 ubiquitin ligase Nedd4‐1. J Neurosci. 2016;36(5):1590‐1595. 10.1523/JNEUROSCI.2964-15.2016 26843640 PMC4737771

[134] Zhang Y , Guo O , Huo Y , Wang G , Man HY . Amyloid‐beta Induces AMPA receptor ubiquitination and degradation in primary neurons and human brains of Alzheimer's Disease. J Alzheimers Dis. 2018;62(4):1789‐1801. 10.3233/JAD-170879 29614651 PMC6353779

[135] Wall MJ , Collins DR , Chery SL , et al. The temporal dynamics of arc expression regulate cognitive flexibility. Neuron. 2018;98(6):1124‐1132.e7. 10.1016/j.neuron.2018.05.012 29861284 PMC6030446

[136] Korb E , Wilkinson CL , Delgado RN , Lovero KL , Finkbeiner S . Arc in the nucleus regulates PML‐dependent GluA1 transcription and homeostatic plasticity. Nat Neurosci. 2013;16(7):874 10.1038/nn.3429 23749147 PMC3703835

[137] Herring BE , Nicoll RA . Long‐term potentiation: from CaMKII to AMPA receptor trafficking. Annu Rev Physiol. 2016;78(1):351‐365. 10.1146/annurev-physiol-021014-071753 26863325

[138] Mauceri D , Cattabeni F , DiLuca M , Gardoni F . Calcium/calmodulin‐dependent protein kinase II phosphorylation drives synapse‐associated protein 97 into spines. J Biol Chem. 2004;279(22):23813 10.1074/jbc.M402796200. 15044483

[139] Hussain NK , Diering GH , Sole J , Anggono V , Huganir RL . Sorting Nexin 27 regulates basal and activity‐dependent trafficking of AMPARs. Proc Natl Acad Sci. 2014;111(32):11840‐11845. 10.1073/pnas.1412415111 25071192 PMC4136608

[140] Chen L , Lau AG , Sarti F . Synaptic retinoic acid signaling and homeostatic synaptic plasticity. Neuropharmacology. 2014;78:3 10.1016/j.neuropharm.2012.12.004 23270606 PMC3884035

[141] Kopeikina KJ , Polydoro M , Tai HC , et al. Synaptic alterations in the rTg4510 mouse model of tauopathy. J Comp Neurol. 2013;521(6):1334‐1353. 10.1002/cne.23234 23047530 PMC3725804

